# Impact of certification status of the institute and surgeon on short-term outcomes after surgery for thoracic esophageal cancer: evaluation using data on 16,752 patients from the National Clinical Database in Japan

**DOI:** 10.1007/s10388-019-00694-9

**Published:** 2019-10-03

**Authors:** Satoru Motoyama, Hiroyuki Yamamoto, Hiroaki Miyata, Masahiko Yano, Takushi Yasuda, Masaichi Ohira, Yoshiaki Kajiyama, Yasushi Toh, Masayuki Watanabe, Yoshihiro Kakeji, Yasuyuki Seto, Yuichiro Doki, Hisahiro Matsubara

**Affiliations:** 1 The Japan Esophageal Society, Tokyo, Japan; 2 Department of Healthcare Quality Assessment, The University of Tokyo Graduate School of Medicine, Tokyo, Japan; 3 The Japanese Society of Gastroenterological Surgery, Tokyo, Japan; 4 Esophageal Surgery, Akita University Hospital, 1-1-1 Hondo, Akita, Japan

**Keywords:** Esophageal cancer, Esophagectomy, Certification, Mortality, Postoperative complication

## Abstract

**Background:**

In 2009, the Japan Esophageal Society (JES) established a system for certification of qualified surgeons as “Board Certified Esophageal Surgeons” (BCESs) or institutes as “Authorized Institutes for Board Certified Esophageal Surgeons” (AIBCESs). We examined the short-term outcomes after esophagectomy, taking into consideration the certifications statuses of the institutes and surgeons.

**Methods:**

This study investigated patients who underwent esophagectomy for thoracic esophageal cancer and who were registered in the Japanese National Clinical Database (NCD) between 2015 and 2017. Using hierarchical multivariable logistic regression analysis adjusted for patient-level risk factors, we determined whether the institute’s or surgeon’s certification status had greater influence on surgery-related mortality or postoperative complications.

**Results:**

Enrolled were 16,752 patients operated on at 854 institutes by 1879 surgeons. There were significant differences in the backgrounds and incidences of postoperative complications and surgery-related mortality rates between the 11,162 patients treated at AIBCESs and the 5590 treated at Non–AIBCESs (surgery-related mortality rates: 1.6% vs 2.8%). There were also differences between the 6854 patients operated on by a BCES and the 9898 treated by a Non-BCES (1.7% vs 2.2%). Hierarchical logistic regression analysis revealed that surgery-related mortality was significantly lower among patients treated at AIBCESs. The institute’s certification had greater influence on short-term surgical outcomes than the operating surgeon’s certification.

**Conclusions:**

The certification system for surgeons and institutes established by the JES appears to be appropriate, as indicated by the improved surgery-related mortality rate. It also appears that the JES certification system contributes to a more appropriate medical delivery system for thoracic esophageal cancer in Japan.

## Introduction

Esophagectomy for thoracic esophageal cancer continues to have high morbidity and mortality rates today [1]. It, therefore, requires a surgeon with experience in the relevant surgical techniques and an institute with a well-trained medical staff providing perioperative care. In 2009, the Japan Esophageal Society (JES) established a certification system for surgeons and institutes to contribute to improving national medical care by enhancing the professional knowledge and skills of esophageal surgeons. A qualified surgeon or institute would be certified as a “Board Certified Esophageal Surgeon” (BCES) or “Authorized Institute for Board Certified Esophageal Surgeon” (AIBCES). The first certification of BCESs and AIBCESs by the JES was in 2010 and 2013, respectively. As of Jan 1, 2019, there were 280 BCESs and 156 AIBCESs nationwide. Using data from the National Database of Hospital-based Cancer Registries in Japan, we recently verified the appropriateness of the certification system for AIBCES by the JES by examining the survival outcomes among patients with thoracic esophageal cancer who underwent an esophagectomy at an AIBCES or a Non-AIBCES [2]. The results revealed that 5-year survival outcomes were better for patients treated at an AIBCES than at a Non-AIBCES. In that report, however, we did not address an important aspect of surgical outcome: morbidity and mortality. In the present study, we analyzed data from the Japanese National Clinical Database (NCD) to compare short-term outcomes between AIBCES and Non-AIBCES. In addition, using a hierarchical multivariable logistic regression analysis, we determined which certification has a greater impact on surgery-related mortality, AIBCES, or BCES.

## Patients and methods

### NCD data registration and study population

Details of the data registration system in the Japanese NCD are available elsewhere [3, 4]. Approximately 1,200,000 cases are registered annually, which corresponds to > 95% of the surgeries performed in Japan. The Japanese Society of Gastroenterological Surgery (JSGS) plays a central role in collecting data on gastroenterological surgeries. Indeed, submitting cases to the present study was a prerequisite for all member institutions of the JSGS. This study investigated patients who underwent esophagectomy for thoracic esophageal cancer, and who were registered in the Japanese NCD between 2015 and 2017. Patients who refused registration in the NCD or who received an emergency operation were excluded. Patients diagnosed as cTx, cT4 or cNx, without data on age, sex, and status on day 30 after esophagectomy were also excluded. The Union for International Cancer Control TNM staging version 7 was adopted to classify pretreatment tumor stage. Surgery-related mortality was defined as a death within 30 days, in or out of the hospital, or an in-hospital death within 90 days after esophagectomy.

### Board Certified Esophageal Surgeon (BCES)

To obtain board certification, a surgeon must accumulate more than 50 surgical points within 5 years. In esophageal cancer surgery, esophagectomy with mediastinal lymph node dissection equals 1 point, while the abdominal operation plus reconstruction using the stomach equals 0.5 points. However, when the colon/jejunum is used for the reconstruction, the operation equals 1 point. Lymph node dissection in the bilateral neck gives 0.5 points. A surgeon must perform more than 15 esophagectomies (thoracic procedure) as minimum requirement within 5 years. In addition, attending JES educational seminars are required.

### Authorized Institute for Board Certified Esophageal Surgeon (AIBCES)

The requirements for becoming an AIBCES are: (1) more than 100 patients diagnosed or treated in-hospital and (2) more than 50 surgeries for esophageal disease within a period of 5 years [2]. That means about ten esophageal surgeries are performed each year. In addition, AIBCESs must employ full-time BCESs, and must also support a training curriculum for board certification.

### Surgery

All esophagectomies for thoracic esophageal cancer with thoracic and abdominal manipulation, with or without cervical manipulation, and concurrent reconstruction were eligible. Both open transthoracic and thoracoscopic esophagectomy (including robot-assisted thoracoscopic esophagectomy) were included.

### Analysis and statistical methods

The software package STATA 15 (STATA Corp., College Station, TX) was used for statistical analyses. Most statistical comparisons between groups were made using the *χ*^2^ test and Fisher’s test, though we compared operation times and estimated blood losses using the Wilcoxon rank-sum test. Values are expressed as the median and 25–75 percentiles or averages. For these comparisons, we first divided the patients into two groups: those who underwent surgery at an AIBCES certified at the time of operation and those who underwent surgery at a Non–AIBCES. We also divided the patients into two other groups: those who underwent surgery by a BCES certified at the time of the operation and those who underwent surgery by a Non–BCES.

Finally, we analyzed the relationship between certification status (AIBCES vs Non–AIBCES, BCES vs Non-BCES) and surgery-related mortality or morbidity. After clustering the patients, hierarchical multivariable logistic regression analysis adjusted for patient-level risk factors was used to analyze its impact on surgery-related mortality and postoperative complications. The patient-level risk factors considered included age at surgery (≤ 59, 60–64, 65–69, 70–74, 75–79, and ≥ 80 years), sex (female vs male), body mass index (BMI; ≤ 25 vs. > 25 kg/m^2^), weight loss (< 10% vs. ≥ 10%), smoking within 1 year (yes vs. no), habitual alcohol use (yes vs. no), any respiratory distress (yes vs. no), preoperative activities of daily living (ADL) with any assistance (yes vs. no), American Society of Anesthesiologists physical status (ASAPS; 1–2 vs. ≥3), diabetes mellitus (DM) with insulin use (yes vs. no), chronic obstructive pulmonary disease (COPD; yes vs. no), hypertension (yes vs. no), congestive heart failure (yes vs. no), previous cardiovascular surgery (yes vs. no), previous cerebrovascular accident (yes vs. no), need for preoperative dialysis (yes vs. no), chronic steroid use (yes vs. no), serum albumin (< 2.5 vs. ≥ 2.5 g/dL), serum creatinine (≤ 1.2 vs>1.2 mg/dL), clinical T stage (T0–Tis–T1, T2–T3), clinical N stage (N0, N1, N2, N3), and use of thoracoscopic surgery (yes vs no). All *P* values were two-sided, and we considered *P* < 0.05 to be statistically significant.

## Results

Enrolled in the study were 16,752 patients operated on at 854 institutes by 1879 surgeons. The annual average numbers of patients at AIBCESs and Non-AIBCESs were 27.8 and 3.7, respectively. The annual average numbers of patients treated by BCESs and Non-BCESs were 13.3 and 3.5, respectively.

### AIBCES vs Non-AIBCES

The numbers of participants receiving esophagectomy at an AIBCES or Non–AIBCES were 11,162 (66.6%) and 5590 (33.4%), respectively (Table 1). There were significant differences between the two groups with respect to age, weight loss, habitual alcohol use, ASAPS grade, DM with insulin use, hypertension, serum albumin, serum creatine, clinical tumor depth (cT), and clinical lymph node metastasis (cN). Patients treated at AIBCSs tended to be in generally better condition, though there were more habitual alcohol users. There were fewer patients with T2–4 tumors treated at AIBCESs, but more with cN1–3 stage disease. At AIBCESs, the operation time was significantly shorter (493 min vs 500 min) with a smaller estimated blood loss (260 ml vs. 290 ml). In the crude data, the incidence of anastomotic leakage was also significantly smaller at AIBCESs (12.9% vs. 15.1%), but the incidence of recurrent laryngeal nerve palsy was greater (13.1% vs. 11.4%). The 30-day mortality rate was significantly lower at AIBCESs than Non-AIBCESs (0.7% vs. 1.1%) in the crude analysis. Likewise, the overall surgery-related mortality rate was significantly lower at AIBCESs than Non-AIBCESs (1.6% vs. 2.8%).

**Table 1:** Background and surgical outcomes in patients received esophagectomy both in AIBCES and Non-AIBCES

	AIBCES (*n* = 11,162)		Non-AIBCES (*n* = 5590)		*P*
Age					< 0.001*
≤ 59	2050	18.4%	866	15.5%	
60–64	1775	15.9%	878	15.7%	
65–69	2804	25.1%	1409	25.2%	
70–74	2461	22.0%	1240	22.2%	
75–79	1564	14.0%	847	15.2%	
≥ 80	508	4.6%	350	6.3%	
Sex					0.261
Female	1973	17.7%	949	17.0%	
Male	9189	82.3%	4641	83.0%	
BMI ≥ 25	1384	12.4%	705	12.6%	0.695
Weight loss ≥ 10%	701	6.3%	490	8.8%	< 0.001*
Smoking within 1 year	4129	37.0%	2059	36.8%	0.842
Habitual alcohol use	7831	70.2%	3356	60.0%	< 0.001*
Respiratory distress	115	1.0%	73	1.3%	0.11
ADL, with any assistance	144	1.3%	86	1.5%	0.193
ASAPS grade ≥3	906	8.1%	546	9.8%	< 0.001*
DM with insulin use	293	2.6%	185	3.3%	0.012*
COPD	947	8.5%	454	8.1%	0.424
Hypertension	4091	36.7%	2210	39.5%	<0.001*
Congestive heart failure	24	0.2%	18	0.3%	0.192
Previous cardiovascular surgery	76	0.7%	38	0.7%	0.994
Previous cerebrovascular accident	93	0.80%	55	1.0%	0.326
Preoperative dialysis	28	0.3%	17	0.3%	0.530
Chronic steroid use	126	1.1%	50	0.9%	0.161
Serum albumin ≤ 2.5 g/dl	60	0.5%	48	0.9%	0.014*
Serum creatine ≥ 1.2 mg/dl	702	6.3%	438	7.8%	< 0.001*
Clinical tumor depth (cT)					0.001*
T0/Tis/T1	4476	40.1%	2089	37.4%	
T2-4	6686	59.9%	3501	62.6%	
Clinical lymph node metastasis (cN)					0.032*
N0	5013	44.9%	2578	46.1%	
N1	3157	28.3%	1520	27.2%	
N2	2167	19.4%	1028	18.4%	
N3	825	7.4%	464	8.3%	
Thoracoscopic esophagectomy	7376	66.1%	3141	56.2%	< 0.001*
Operation time (min)^a^	493	(405–588)	500	(413-592)	0.013*
Blood loss (ml)^a^	260	(127–473)	290	(140–530)	< 0.001*
Anastomotic leakage	1445	12.9%	846	15.1%	<0.001*
Postoperative pneumonia	1551	13.9%	772	13.8%	0.881
Recurrent laryngeal nerve palsy	1461	13.1%	640	11.4%	0.003*
30-day mortality	78	0.7%	62	1.1%	0.006*
Surgery-related mortality	179	1.6%	154	2.8%	< 0.001*

*AIBCES* Authorized Institute for board certified esophageal surgeon, *BMI* body mass index, *ADL* activities of daily living, *ASAPS* American Society of Anesthesiologists Physical Status, *DM* diabetes mellitus, *COPD* chronic obstructive pulmonary disease

*Significant difference

^a^Analyzed using Wilcoxon rank-sum test

### BCES vs Non-BCES

The numbers of patients treated with esophagectomy by a BCES or Non–BCES were 6854 (40.9%) and 9898 (59.1%), respectively (Table 2). There were significant differences between the two groups with respect to age, weight loss, habitual alcohol use, hypertension, previous cerebrovascular accident, preoperative dialysis, serum creatine, cT, and cN. Patients operated on by BCESs tended to be in generally better condition, though there were a greater number of habitual alcohol users. For BCESs, operation time was significantly longer (501 min vs. 492 min), but the estimated blood loss was smaller (260 ml vs. 277 ml). The difference in these factors between BCESs and Non-BCESs was smaller than between AIBCESs and Non-AIBCESs. In the crude analysis, the incidence of anastomotic leakage was significantly smaller with BCESs than Non-BCESs (13.2% vs. 14.0%), but the difference in the incidences was smaller than the difference in those rates between AIBCES and Non-AIBCES. Although the 30-day mortality rates did not differ between the two groups (0.7% vs. 0.9%), the overall surgery-related mortality rates were significantly lower with BCESs than Non-BCESs (1.7% vs. 2.2%) in the crude analysis. However, the difference in surgery-related mortality between BCESs and Non-BCESs was smaller than between AIBCESs and Non-AIBCESs.

**Table 2:** Background and surgical outcomes in patients treated with esophagectomy by a BCES or Non-BCES

	BCES (*n* = 6854)		Non-BCES (*n *= 9898)		*P*
Age					0.003*
≤ 59	1246	18.2%	1670	16.9%	
60–64	1132	16.5%	1521	15.4%	
65–69	1720	25.1%	2493	25.2%	
70–74	1487	21.7%	2214	22.4%	
75–79	961	14.0%	1450	14.6%	
≥ 80	308	4.5%	550	5.6%	
Sex					0.917
Female	1193	17.4%	1729	17.5%	
Male	5661	82.6%	8169	82.5%	
BMI ≥ 25	835	12.2%	1254	12.7%	0.349
Weight loss ≥ 10%	406	5.9%	785	7.9%	< 0.001*
Smoking within 1 year	2570	37.5%	3618	36.6%	0.213
Habitual alcohol use	4858	70.9%	6329	63.9%	< 0.001*
Respiratory distress	64	0.9%	124	1.3%	0.054
ADL, with any assistance	80	1.2%	150	1.5%	0.057
ASAPS grade ≥ 3	573	8.4%	879	8.9%	0.239
DM with insulin use	179	2.6%	299	3.0%	0.118
COPD	581	8.5%	820	8.3%	0.658
Hypertension	2501	36.5%	3800	38.4%	0.012*
Congestive heart failure	19	0.3%	23	0.2%	0.568
Previous cardiovascular surgery	52	0.8%	62	0.6%	0.306
Previous cerebrovascular accident	44	0.6%	104	1.1%	0.005*
Preoperative dialysis	9	0.1%	36	0.4%	0.004*
Chronic steroid use	75	1.1%	101	1.0%	0.645
Serum albumin ≤ 2.5 g/dl	37	0.5%	71	0.7%	0.158
Serum creatine ≥ 1.2 mg/dl	398	5.8%	742	7.5%	< 0.001*
Clinical tumor depth (cT)					0.034*
T0/Tis/T1	2752	40.2%	3813	38.5%	
T2–4	4102	59.8%	6085	61.5%	
Clinical lymph node metastasis (cN)					0.038*
N0	3064	44.7%	4527	45.7%	
N1	1993	29.1%	2684	27.1%	
N2	1292	18.9%	1903	19.2%	
N3	505	7.4%	784	7.9%	
Thoracoscopic esophagectomy	4596	67.1%	5921	59.8%	< 0.001*
Operation time (min)^a^	501	(413–592)	492	(405–588)	0.001*
Blood loss (ml)^a^	260	(130–470)	277	(130–510)	< 0.001*
Anastomotic leakage	907	13.2%	1384	14.0%	0.165
Postoperative pneumonia	957	14.0%	1366	13.8%	0.766
Recurrent laryngeal nerve palsy	830	12.1%	1271	12.8%	0.160
30-day mortality	50	0.7%	90	0.9%	0.209
Surgery-related mortality	114	1.7%	219	2.2%	0.012*

*AIBCES* Authorized Institute for board certified esophageal surgeon, *BMI* body mass index, *ADL* activities of daily living, *ASAPS* American Society of Anesthesiologists Physical Status, *DM* diabetes mellitus, *COPD* chronic obstructive pulmonary disease

*Significant difference

^a^Analyzed using Wilcoxon rank-sum test

### Surgery-related mortality and postoperative complications among four categories AIBCES/Non-AIBCES and BCES/Non-BCES

Table 3 shows the incidences of surgery-related mortality and postoperative complications among the four categories defined by certification type. The surgery-related mortality rate was highest in the Non-AIBCES/Non-BCES group (2.9%) and lowest in the AIBCES/Non-BCES group (1.5%) in the crude analysis. The incidence of anastomotic leakage was highest in the Non-AIBCES/Non-BCES group (15.7%) and lowest in the Non-AIBCES/BCES group (11.5%). The incidence of postoperative pneumonia and recurrent laryngeal nerve palsy was lowest in the Non-AIBCES/BCES group (10.5% and 9.7%). In summary, the incidence of postoperative complications was lowest in the Non-AIBCES/BCES group.

**Table 3:** Surgery-related mortality and postoperative complications among four categories of AIBCES/Non-AIBCES and BCES/Non-BCES

	Non-AIBCES/Non-BCES 4816 (28.7%)		Non-AIBCES/BCES 774 (4.6%)		AIBCES/Non-BCES 5082 (30.3%)		AIBCES/BCES 6080 (36.3%)	
Surgery-related mortality	141	2.9%	13	1.7%	78	1.5%	101	1.7%
Anastomotic leakage	757	15.7%	89	11.5%	627	12.3%	818	13.5%
Postoperative pneumonia	691	14.3%	81	10.5%	675	13.3%	876	14.4%
Recurrent laryngeal nerve palsy	565	11.7%	75	9.7%	706	13.9%	755	12.4%

*AIBCES* Authorized Institute for board certified esophageal surgeon, *BCES* board certified esophageal surgeon

### Hierarchical multivariable logistic regression analysis adjusted for patient-level risk factors

**Fig. 1:**
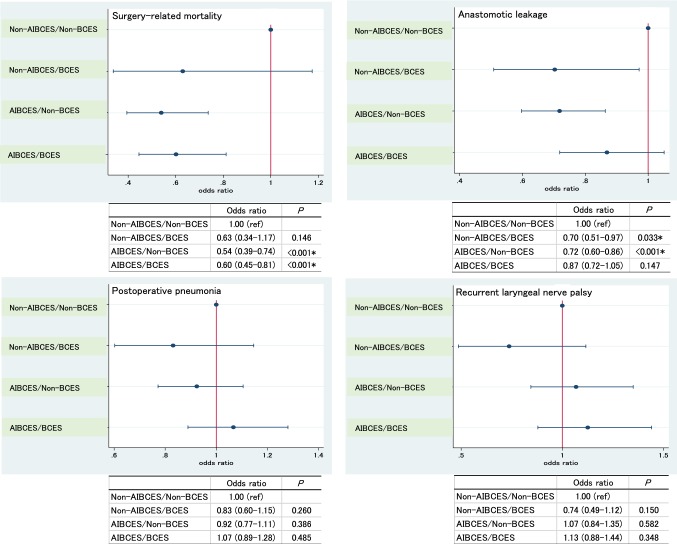
Results of a hierarchical multivariable logistic regression analysis of the incidence of surgery-related mortality and operative complications (anastomotic leakage, postoperative pneumonia and recurrent laryngeal nerve palsy) adjusted for patient-level risk factors in the indicated four categories

Figure 1 shows the results of a hierarchical multivariable logistic regression analysis of the incidences of surgery-related mortality and postoperative complications adjusted for patient-level risk factors in the four categories defined by certification type. Surgery-related mortality was significantly lower in patients in the AIBCES/Non-BCES (odds ratio (OR): 0.54, 95% CI: 0.39–0.74, *P* < 0.001) and AIBCES/BCES groups (OR: 0.60, 95% CI: 0.45–0.81, *P* < 0.001) than in the Non-AIBCES/Non-BCES group. This result indicates that the certification of the institute, not the surgeon, is the stronger factor affecting surgery-related mortality. The incidence of anastomotic leakage was significantly lower among patients in the Non-AIBCES/BCES (OR: 0.70, 95% CI: 0.51–0.97, *P* = 0.033) and AIBCES/Non-BCES (OR: 0.72, 95% CI: 0.60–0.86, *P* < 0.001) groups than in the Non-AIBCES/Non-BCES group. This result shows that the certification of both the institute and surgeon is strong factors affecting the incidence of anastomotic leakage. On the other hand, the incidence of postoperative pneumonia and recurrent laryngeal nerve palsy did not differ among the four groups, though the incidence of recurrent laryngeal nerve palsy tended to be higher in the AIBCS groups (OR: 1.07, 95% CI: 0.84–1.35, *P* = 0.582, OR: 1.13, 95% CI: 0.88–1.44, *P* = 0.348) and to be lower in Non-AIBCES/BCES group (OR: 0.74, 95% CI: 0.49–1.12, *P* = 0.150) than in the Non-AIBCES/Non-BCES group.

## Discussion

This study produced several noteworthy results. First, 29% of patients received esophagectomy for thoracic esophageal cancer at a Non-AIBCES by a Non-BCES in Japan. Second, patients with more severe conditions were more frequently operated on at Non-AIBCSs than AIBCES and more frequently by Non-BCESs than BCESs. Third, hierarchical multivariable logistic regression analysis of the incidence of surgery-related mortality adjusted for patient-level risk factors revealed that the surgery-related mortality rate was significantly lower in the AIBCES/Non-BCES (OR: 0.54) or AIBCES/BCES (OR: 0.60) group than in the Non-AIBCES/Non-BCES group. The difference in surgery-related mortality between BCESs and Non-BCESs was smaller than between AIBCESs and Non-AIBCESs in the crude analysis. Fourth, the certification of both the institute and surgeon is strong factors affecting the incidence of anastomotic leakage, but the incidence of postoperative complications (pneumonia and recurrent laryngeal nerve palsy) did not differ among the four groups in the hierarchical multivariable logistic regression analysis.

Despite recent advances in surgical techniques and perioperative management, esophagectomy remains a highly invasive surgery associated with a high mortality rate and potentially fatal postoperative complications [1]. Several studies have demonstrated a strong association between operative volume (hospital volume) and surgery-related mortality [5–9]. Nishigori et al. reported that the unadjusted operative mortality rate in hospitals performing 30 or more procedures per year was more than 3 times lower than in hospitals performing fewer than ten procedures annually [6]. Yoshida et al. reported that the most recent data in the NCD showed that a lower annual hospital volume of esophagectomies was a significant independent risk factor for surgery-related mortality [4]. The OR for hospitals with an esophagectomy volume of 18.4–43 per year was 0.43 compared to hospitals with a volume of 0.2–6.2 per year, and was 0.65 for hospitals performing 6.4–18.2 esophagectomies per year. These reports indicate that the centralization of esophageal surgery in Japan, such that hospitals perform more than 20–30 esophagectomies per year is most appropriate, as judged by the present social and medical situations of our country. One provision to be AIBCES is more than 50 surgeries for esophageal disease within a period of 5 years. This means that the minimum number of esophagectomies for esophageal cancers at an AIBCES is less than 10 per year, which is fewer than reported in the above reports. However, the actual number of esophagectomies per year at AIBCESs is larger. In this study, the average number of surgeries per year at AIBCESs was 27.8, as opposed to 3.7 at Non-AIBCES.

Why was the post‐esophagectomy mortality rate lower at AIBCESs than at Non‐AIBCESs? Ghaferi et al. reported that surgery-related mortality reflects the ability to avoid mortality from severe complications; that is, high–volume esophagectomy centers are less likely to exhibit “failure to rescue.” [10]. Other aspects of care associated with improved outcomes include the abilities of the nursing and staffing care teams [11]. In the present study, the incidence of anastomotic leakage was lower at AIBCESs, but that of recurrent laryngeal nerve palsy was higher. The incidence of postoperative pneumonia, which can lead to surgery-related mortality, was equal between AIBCESs and Non-AIBCESs. This demonstrates that the incidence of postoperative complications is not directly associated with surgery-related mortality. Recently, there have been reports, suggesting that the disparity in outcomes after complex surgery may not be due to differences in hospital volume, but to differences in the patients’ characteristics [12, 13]. Indeed, in our data, many of the patient’s clinical characteristics were significantly worse (high age, weight loss ≥ 10%, ASAPS ≥ 3, DM with insulin use, hypertension, serum albumin < 2.5 g/dl, and serum creatinine > 1.2 mg/dl) in patients treated at Non-AIBCESs. It was evident that a study with adjustments for risk and cancer stage was needed before drawing any conclusions. In the present study, therefore, we performed risk‐adjusted analyses, which led to the same conclusion.

The Japanese Society of Hepato‐Biliary‐Pancreatic Surgery (JSHBPS) established a board certification system for expert surgeons in the hepato‐biliary‐pancreatic (HBP) field and began certifying expert surgeons in 2008. Only two societies in Japan, the JES and JSHBPS, have established a board certification system for specialists with a high level of expertise in their field, which is often referred to as the “third floor.” For esophageal and HBP surgeons, it is first possible to be certified as a “Board Certified Surgeon” by the Japan Surgical Society (1st floor), then as a “Board Certified Surgeon in Gastroenterology” by the JSGS (2nd floor). A BECS is a specialist referred to as the third floor. In 2016, Miura et al. reported that the operative mortality rates after hepatectomies performed with participation of certified instructors or expert surgeons were significantly better than when this operation was performed without them (3.5% vs. 4.3%) [14]. Likewise, operative mortality rates were lower after pancreaticoduodenectomies performed with participation of certified instructors or expert surgeons (2.2% vs. 3.8%) [15]. This result is consistent with the earlier findings of McKay et al. [16], who reported that the 90‐day in‐hospital mortality rate after hepatic resection was 4.6% when performed by surgeons with HBP fellowship training and with a full year of training in HBP surgery, but was 6.3% when performed by surgeons with a surgical oncology fellowship, 7.2% when performed by surgeons with other subspecialty training, and 15.3% when performed by general surgeons.

In our data, although there was not a significant difference in the incidences of postoperative complications between esophagectomy by a BCES and Non-BCES, the surgery-related mortality rates did significantly differ (1.7% vs. 2.2%). However, the backgrounds of the patients treated were also different. Surprisingly, patients treated by BCESs were in better condition than those treated by Non-BCESs. This may be because a large fraction of the operations performed by BCESs are at AIBCESs, where patient selection is stricter than at Non-AIBCESs. This made risk‐adjusted hierarchical multivariable logistic regression analysis necessary. This analysis revealed that whether the surgeon was or was not a BCES did not directly influence surgery-related mortality. There is thus a difference between our findings and those previously reported by Miura et al. for HBP. Contributing to this difference is first the different affected organs (esophagus vs HBP). Second, Miura divided the patients based on participation of a surgeon certified in the HBP field, whereas we divided them based on whether the surgeon performing the operation was a BCES or Non-BCES. Consequently, the size of the BCES group was smaller than the Non-BCES group (6854 vs 9898). However, it is likely that even in the Non-BCES group, there was frequent participation of a BCES, particularly at AIBCESs [17]. In esophageal cancer surgery, the operation consists of resection in three fields (neck, thorax and abdomen) and reconstruction from the neck to the abdomen. The operation in the neck and abdomen is usually performed simultaneously in a high-volume hospital. In fact, there are multiple operating surgeons at work during esophageal cancer surgery. This is not registered directly in the NCD system, which permits only one operating surgeon to be listed. Third, we performed hierarchical multivariable logistic regression analysis that included not only the patients’ backgrounds, but also factors related to the institutions and surgeons, which Miura et al. did not. We think that it is for these reasons that factors related to the operating surgeon did not strictly influence postoperative complications.

Although it is clear from earlier studies that centralization of esophagectomy to high-volume hospitals improves short-term surgical results in patients with esophageal cancer, such centralization can cause problems, especially in local regional areas away from the cities. Surgery at a hospital far from the area where the patient lives is not suitable because of the characteristics of esophageal cancer. Esophageal cancer surgery is highly stressful for patients and requires intensive postoperative management. Moreover, esophagectomy requires pre- and postoperative adjuvant treatments. Consequently, the period of treatment is long. To achieve a complete cure, these patients require family and/or social support during recovery from the initial treatment and during the long-term follow-up. It is, therefore, necessary to consider how to balance centralization and equalization so as to optimize the medical delivery system not only for metropolitan areas but also for local regional areas away from the city. This research shows that the AIBCES certification system provided by the JES is fully functioning and maintained at a high level for esophageal cancer surgical treatment. There are differences in the best medical treatment flow for different diseases in different countries. There, too, medical societies for each specialized field establish appropriate medical delivery systems, and facility certification systems appear to play an important role, just as with the JES.

Despite the highly reliable data acquired via a robust statistical analysis of a very large cohort, this study has several limitations. First, there were differences among the esophagectomies registered in the NCD; that is, there were differences in the extent and intensity of lymph node dissection as well as in the route and organs used for reconstruction. Indeed, the incidence of recurrent laryngeal nerve palsy was higher in AIBCESs than Non-AIBCES (13.1% vs 11.4%). This may indicate a difference in the intensity of the bilateral cervical and upper mediastinal lymph node dissection between the two groups. Second, preoperative adjuvant therapy was not included in the risk factor adjustment. Using the NCD, Yoshida et al. showed that the surgery-related mortality was significantly higher in patients who received preoperative chemoradiotherapy than in those receiving only chemotherapy or no preoperative treatment [4]. In our earlier study, the fraction of patients receiving preoperative chemoradiotherapy were much smaller in Non-AIBCESs. Furthermore, Tsukada et al. reported that patients at lower volume hospitals were less likely to receive neoadjuvant therapy for esophageal cancer [18]. This tendency may influence the result of this study. Third, there is the possibility that unmeasured confounders influenced the results of these analyses.

In conclusion, our findings indicate that the institute certification system established by the JES is appropriate, as indicated by improved surgery-related mortality. Together with the surgeon and institute certification system, the JES is contributing to a more appropriate medical delivery system for thoracic esophageal cancer in Japan.
